# Phenolic Concentrations and Antioxidant Properties of Wines Made from North American Grapes Grown in China

**DOI:** 10.3390/molecules17033304

**Published:** 2012-03-14

**Authors:** Lei Zhu, Yali Zhang, Jiajin Deng, Huirong Li, Jiang Lu

**Affiliations:** 1 College of Food Science and Nutritional Engineering, China Agricultural University, Beijing 100083, China; Email: Zhulei610@gmail.com (L.Z.); olivia.yl.zhang@gmail.com (Y.Z.); jiajin.deng@gmail.com (J.D.); lihuirong173@yahoo.cn (H.L.); 2 Center for Viticulture and Small Fruit Research, Florida Agricultural and Mechanical University, Tallahassee, FL 32317, USA

**Keywords:** wine, North American grapes, phenolic concentration and composition, antioxidant capacity

## Abstract

The characteristics of wine phenolics found in several North American and (for comparison) European grape cultivars grown in China were analyzed. This was done to find non-*Vitis* *vinifera* wines with prominent features in order to diversify the kinds of wines. The phenolic richness and antioxidant activity decreased in the order: red > rose > white wines. In the red wines, the American grape ‘Cynthiana’ had the highest total concentrations of phenols, anthocyanins, flavonols and phenolic acids, as well as antioxidant capacity, followed by the French hybrid ‘Chambourcin’, the lowest were detected in two European grape varieties, ‘Merlot’ and ‘Cabernet Sauvignon’, while the total flavon-3-ols levels were reversed among these red grape cultivars. The highest concentration of stilbenes out of all the wines analyzed was found in the ‘Merlot’ variety. There were significant differences among wine phenolic compositions between North American and European grape cultivars. The antioxidant activities were significantly related to the concentrations of total phenols (r^2^ = 0.996), anthocyanins (r^2^ = 0.984), flavonols (r^2^ = 0.850) and gallic acid (r^2^ = 0.797). The prominent features of wine aroma and nutrition could make the American grape wines attractive to consumers. It is therefore necessary to perform further research on cultural practices and wine making involving these grapes.

## 1. Introduction

Phenolic compounds are the most abundant secondary metabolites in plants, and also exist in the everyday diet of humans. These compounds are efficient antioxidants and may help prevent degenerative processes such as cancer, cardiovascular disease and diabetes, among others [1,2]. As a result, many foods which contain abundant phenolic compounds have attracted increased consumer attention in recent years. Researchers in the field of food and nutrition have investigated the contents and compositions of the phenolic constituents in these foods by chemical methods and instrumental analysis, and studied the bioactivities of the phenolics both *in vitro* and *in vivo*. For example, onions were found to be rich in flavonols (90–360 mg quercetin/kg fresh weight for total contents), and anthocyanins were also present in red varieties (3–29 mg cyanidin/kg fresh weight for total contents) [3,4,5]; anthocyanins were the main phenolics present in the fresh mulberries (2,300–3,000 mg cyanidin/kg fresh weight for total contents), which chiefly contributed to the total antioxidant activity of mulberries [6]; anthocyanins (80–2,400 mg cyanidin/kg fresh weight) [7], flavonols (25–197 mg/kg fresh weight in skins) [8,9] and flavan-3-ols (9–96 mg/kg fresh weight in skins and 330–1,390 mg/kg fresh weight in seeds) [9] were the major kinds of phenolic compounds in the grapes, and there was a range of 2,000–4,250 mg gallic acid/kg fresh weight for total phenolic contents present in the edible parts (skins and pulps), which had high antioxidant and antiproliferative activities [7]. 

Wines made from grapes are also one of the most important sources of phenolic compounds (160–3200 mg gallic acid/L for total phenolic contents) [10,11,12], and are recommended for moderate consumption as a alcoholic beverage according to the reports on the so-called French Paradox [13]. Not only the nutritional benefits but also the quality of wine largely depend on the composition and contents of its phenolic compounds, so phenolic analysis can be used as an effective tool in characterizing different wines [14,15,16,17]. According to previous reports, wine phenolics can be divided into five groups: (i) phenolic acids which consist of hydroxybenzoic and hydroxycinnamic acids that contribute to the colour of red/rose wines as copigments of anthocyanins [18,19,20]; (ii) stilbenes, mainly referring to resveratrol and its glycosides; (iii) anthocyanins, which are responsible for the colour of red/rose wines [21]; (iv) flavan-3-ols containing monomeric catechins and proanthocyanidins that are the main phenolic compounds involved in the astringency, bitterness and structure of wines, and are also an important factor in stabilizing the colour of aging wines as anthocyanin copigments [22,23,24,25]; and finally, (v) flavonols which play an important role in bitterness, and also act as copigments of anthocyanins [26,27].

The Chinese wine industry has grown very rapidly in the 21st century. Wine production has increased about 18% annually during the last decade, reaching some 960,000 tons in 2009. Even so, there is still great room for the industry and market to grow. Unfortunately, almost all wine grape varieties are limited to a few traditional *Vitis vinifera* L. or so-called European grape cultivars such as ‘Cabernet Sauvignon’, ‘Merlot’, and ‘Chardonnay’ although a few Chinese cultivars derived from *V. amurensis* and its hybrids exist in the wine-growing regions of northeast China. Studies on the adaptations of other wine grapes such as the North American grapes under Chinese climate conditions are rather rare, and to the best of our knowledge, the phenolic features of the wines made from North American grapes grown in China have not yet been characterized. The objective of this study is to investigate and analyze the phenolic compositions and antioxidant activity of the wines made from North American grapes grown in China, and to compare the phenolic characteristics of these wines with two traditional European wines.

## 2. Results

### 2.1. Oenological Parameters

The descriptive statistics for the berries and wines of the seven grape cultivars are given in Table 1. For the grape berries, in general, the North American grapes had relatively heavier berry weights (2.36 g in average), lower total soluble solids (TSS, 195 g/L in average) and higher titrable acidity (TA, 5.9 g/L in average) than the two European grapes (1.37 g, 213 g/L and 3.7 g/L in average). 

The berry weight (1.03 g) and the TSS (238 g/L) of the American grape ‘Cynthiana’ were the exceptions. As for the wines, corresponding with the TSS and TA of the grape berries, the wines made from the American grapes possessed lower alcoholic strengths (11.5% in average) and residual sugars (RS, 0.5 g/L in average) and higher TA (6.8 g/L in average) than the two European wines (12.3%, 1.7 g/L and 0.32 g/L in average), except for the wine from ‘Cynthiana’ (alcohol: 13.7%; RS: 2.2 g/L). There were no significant differences among the volatile acidities (VA) of the wine samples (0.2–0.4 g/L).

### 2.2. Total Phenolic Concentrations

The results of the determination of total phenolic concetrations (TPC) in the wine samples are presented in Table 2. In general, the TPC of red wines was significantly higher than that observed in white wines, and TPC in the rose wine lay in between.

The red wine from the American cultivar ‘Cynthiana’ had the highest TPC (939.67 mg GAE/L), followed by the red wines from the French hybrid ‘Chambourcin’ (597.17 mg GAE/L), the European grapes ‘Cabernet Sauvignon’ (434.67 mg GAE/L) and ‘Merlot’ (398.00 mg GAE/L). The TPC in the rose wine of north American *V. labrusca* ‘Catawba’ was 368.83 mg GAE/L. While in the two white wines, the *V. labrusca* ‘Noah’ (312.17 mg GAE/L) had obviously higher TPC than the French hybrid ‘Villard Blanc’ (224.67 mg GAE/L).

### 2.3. Phenolic Compositions

In order to better expound the wine penolic profiles, the phenolic compounds in these seven wines were assayed and characterized by HPLC–ESI-MS/MS. The phenolic compounds could be divided into five categories, including anthocyanins, flavonols, flavan-3-ols, phenolic acids and stilbenes. The detailed profiles in each of these wines are shown in Table 3 and Table 4.

**Table 1:** The general characteristics of berries and wines of different grape cultivars.

Cultivars	Species	Grape			Wine			
		Weight (g)	TSS (g/L)	TA (g/L)	Alcohol (% vol)	TA (g/L)	VA (g/L)	RS (g/L)
Merlot (ML)	*V. vinifera*	1.28 ± 0.29 ^a^	211 ± 7 ^b^	3.6 ± 0.2 ^a^	12.2 ± 0.4 ^b^	4.5 ± 0.2 ^a^	0.35 ± 0.05	1.7 ± 0.1 ^b^
Cabernet Sauvignon (CS)	*V. vinifera*	1.45 ± 0.35 ^ab^	214 ± 5 ^b^	3.8 ± 0.3 ^a^	12.3 ± 0.3 ^b^	4.6 ± 0.1 ^a^	0.29 ± 0.04	1.7 ± 0.2 ^b^
Chambourcin (CB)	*V. rupestris*, *V. lincecumii, V. vinifera*	2.41 ± 0.42 ^bc^	195 ± 5 ^a^	4.2 ± 0.3 ^a^	11.5 ± 0.2 ^a^	5.1 ± 0.3 ^a^	0.39 ± 0.06	0.6 ± 0.1 ^a^
Cynthiana (CT)	*V. aestivalis* (or American *Vitis*, *V. vinifera*)	1.03 ± 0.21 ^a^	238 ± 7 ^c^	5.7 ± 0.2 ^b^	13.7 ± 0.5 ^c^	6.6 ± 0.2 ^b^	0.35 ± 0.03	2.2 ± 0.2 ^b^
Catabaw (CA)	*V. labrusca* (or *V. labrusca*, *V. vinifera*)	2.78 ± 0.39 ^c^	197 ± 5 ^a^	6.1 ± 0.3 ^bc^	11.6 ± 0.3 ^a^	7.0 ± 0.2 ^c^	0.27 ± 0.04	0.4 ± 0.2 ^a^
Villard Blanc (VB)	*V. labrusca*, *V. vinifera*	2.24 ± 0.41 ^bc^	194 ± 5 ^a^	6.8 ± 0.2 ^c^	11.5 ± 0.3 ^a^	7.6 ± 0.1 ^d^	0.31 ± 0.05	0.5 ± 0.2 ^a^
Noah (NO)	*V. labrusca*	2.02 ± 0.33 ^b^	192 ± 6 ^a^	6.9 ± 0.2 ^c^	11.5 ± 0.2 ^a^	7.7 ± 0.1 ^d^	0.36 ± 0.03	0.4 ± 0.1 ^a^

Values are means of duplicate determination ± S.D. Diffrent letters in each column are significantly different at the 0.05 level according to ANOVA.

**Table 2:** The total phenolic concentrations and antioxident capacities in the wines from different grape cultivars.

Cultivars	Wine types	Total phenolic content (mg GAE/L)	Ferric reducing antioxidant power (mmol TE/L)
ML	Red	398.00 ± 4.71 ^cd^	2.91 ± 0.14 ^cd^
CS	Red	434.67 ± 11.79 ^d^	3.35 ± 0.11 ^d^
CB	Red	597.17 ± 15.32 ^e^	5.18 ± 0.20 ^e^
CT	Red	939.67 ± 25.93 ^f^	8.47 ± 0.28 ^f^
CA	Rose	368.83 ± 3.54 ^c^	2.72 ± 0.04 ^bc^
VB	White	224.67 ± 2.36 ^a^	1.35 ± 0.04 ^a^
NO	White	312.17 ± 8.25 ^b^	2.17 ± 0.06 ^b^

The abbreviations of the cultivars are the same as Table 1. Values are means of duplicate determination ± S.D. Diffrent letters in each column are significantly different at the 0.05 level according to ANOVA.

**Table 3:** Retention times, ESI-MS/MS *m/z* values (molecular ion (MS); product ions (MS^2^)) and concentrations of individual anthocyanins identified in the red and rose wines from different grape cultivars.

Peak	Compound	Rt (min)	MS; MS^2^ (*m/z*)	Red wines				Rose wine
				ML	CS	CB	CT	CA
1	Dp-3,5-diglc	2.85	627;465,303	nd	nd	nd	12.93 ± 4.05	1.88 ± 0.17
2	Cy-3,5-diglc	3.10	611;449,287	nd	nd	nd	5.22 ± 1.88	2.03 ± 0.22
3	Dp-3-glc	4.05	465;303	nd	nd	nd	6.81 ± 1.47	1.40 ± 0.36
4	Pt-3,5-diglc	4.41	641;479,317	nd	nd	6.10 ± 1.23	8.39 ± 2.13	nd
5	Cy-3-*O*-glc	5.01	449;287	nd	nd	nd	nd	1.50 ± 0.23
6	Pn-3,5-diglc	5.8	625;463, 301	nd	nd	nd	22.32 ± 1.63	nd
7	Mv-3,5- diglc	6.02	655;493, 331	nd	nd	50.55 ± 3.07	69.64 ± 2.59	nd
8	Pt-3-glc	7.27	479;317	nd	nd	4.09 ± 1.67	2.71 ± 1.16	nd
9	Pn-3-glc	10.00	463;301	nd	nd	nd	2.91 ± 1.86	nd
10	Mv-3-glc	10.80	493;331	3.19 ± 1.60	5.12 ± 1.83	7.29 ± 3.38	8.33 ± 2.21	tr
11	Pt-3-glc-acetaldehyde	12.42	503	nd	nd	4.03 ± 1.43	nd	nd
12	Dp-3-acglc-5-glc	13.43	773;611,465,303	nd	nd	nd	5.23 ± 1.76	nd
13	Mv-3-acglc-5-glc	14.46	697;655,493,331	nd	nd	2.07 ± 1.12	nd	nd
14	Pn-3-glc-acetaldehyde	15.39	531	1.46 ± 0.22	nd	nd	nd	nd
15	Mv-3-glc-pyruvic acid	15.71	561;399	3.18 ± 0.86	2.11 ± 0.32	2.21 ± 0.41	1.91 ± 0.51	tr
16	Mv-3-glc-acetaldehyde	16.65	517;355	1.32 ± 0.05	1.53 ± 0.37	1.51 ± 0.23	2.29 ± 1.36	nd
17	Mv-3-acglc-pyruvic acid	17.48	603;399	1.74 ± 0.36	2.78 ± 0.69	nd	nd	nd
18	Mv-3-acglc-acetaldehyde	18.29	559;355	1.33 ± 0.13	1.65 ± 0.36	nd	nd	nd
19	Cy-3-cmglc-5-glc	18.63	757;595,449,287	nd	nd	nd	5.10 ± 1.79	nd
20	Mv-3-glc-ethyl-(epi)catechin	18.99	809	1.84 ± 0.14	1.81 ± 0.45	nd	nd	nd
21	Pt-3-cmglc-5-glc	19.32	787;625,479,317	nd	nd	1.99 ± 0.49	7.65 ± 1.78	nd
22	Dp-3-cmglc	20.87	611;303	nd	nd	nd	2.06 ± 0.31	nd
23	Mv-3-acglc	21.68	535;331	1.98 ± 0.42	3.76 ± 0.86	nd	nd	nd
24	Cy-3-cmglc	22.67	595;287	nd	nd	nd	1.28 ± 0.15	nd
25	Mv-3-cmglc-5-glc	23.10	801;639,493,331	nd	nd	3.26 ± 1.66	18.36 ± 0.89	nd
26	Mv-3-cmglc-pyruvic acid	24.21	707;349	1.32 ± 0.06	nd	nd	nd	nd
27	Pt-3-cmglc	25.93	625;317	nd	nd	nd	1.19 ± 0.23	nd
28	Pn-3-cmglc	27.00	609;301	1.17 ± 0.03	1.71 ± 0.74	nd	nd	nd
29	Mv-3-cmglc	29.49	639;331	1.39 ± 0.16	1.58 ± 0.55	1.50 ± 0.06	2.40 ± 1.06	nd
30	Mv-3-glc-4-vinyl(epi)catechin	30.09	805	1.24 ± 0.03	nd	nd	nd	nd
31	Mv-3-glc-4-vinylphenol	31.92	609;447	nd	1.14 ± 0.18	nd	nd	nd
32	Mv-3-glc-4-vinylguaiacol	32.83	639;477	nd	nd	nd	nd	nd
33	Mv-3-acglc-4-vinyl-(epi)catechin	34.02	847;439	1.23 ± 0.03	1.50 ± 0.41	nd	nd	nd
	&otal			22.38 ± 0.98 ^b^	24.70 ± 6.39 ^b^	84.59 ± 2.46 ^c^	186.75 ± 8.54 ^d^	6.81 ± 0.08 ^a^

The abbreviations of the cultivars are the same as Table 1. Values are means of duplicate determination ±S.D. nd means not detected. tr means trace. Abbreviations: dp, delphinidin; cy, cyanidin; pt, petunidin; pn, peonidin; mv, malvidin; diglc, diglucoside; glc, glucoside; acglc, (6-acetyl)-glucoside; cmglc, (6-coumaroyl)-glucoside. Diffrent letters in each row of total concetrations are significantly different at the 0.05 level according to ANOVA.

**Table 4:** Retention times, ESI-MS/MS *m/z* values (molecular ion (MS); product ions (MS^2^)) and concentrations of individual non-anthocyanin phenolic compounds identified in the wines from different grape cultivars.

Peak	Compound	Rt (min)	MS; MS^2^ (*m/z*)	Red wines				Rose wine	White wines	
				ML	CS	CB	CT	CA	VB	NO
**Dihydroflavonols and flavonols (mg QE /L)**										
1	Dq-3-xyl	12.43	435;303	1.15 ± 0.81	nd	1.27 ± 0.47	2.85 ± 2.00	2.39 ± 1.27	nd	0.80 ± 0.47
2	Dq-3-hex	13.97	465;303	0.40 ± 0.05	0.72 ± 0.67	1.11 ± 0.77	nd	2.35 ± 1.31	nd	nd
3	Dq	14.90	303	1.23 ± 0.38	1.85 ± 0.70	0.62 ± 0.47	0.66 ± 0.36	1.28 ± 0.78	nd	nd
4	Dq-3-gcn	16.23	479;303	2.78 ± 0.92	3.41 ± 0.99	2.40 ± 1.22	2.61 ± 0.92	1.87 ± 0.77	nd	nd
5*	Q-3-caglc	16.98	625;301	nd	nd	2.63 ± 1.20	nd	nd	nd	nd
6	Q	17.06	301	nd	nd	5.63 ± 0.77	6.08 ± 0.91	1.04 ± 0.47	3.31 ± 2.41	2.37 ± 1.2
7	Dq-3-rha	17.73	449;303	3.96 ± 1.22	2.52 ± 0.46	0.98 ± 0.81	2.42 ± 0.90	0.91 ± 0.37	nd	nd
8	Q-3-gal	18.08	463;301	nd	nd	nd	1.61 ± 0.88	nd	nd	nd
9	Q-3-glc	18.64	477;301	nd	0.84 ± 0.51	3.22 ± 1.56	2.95 ± 0.57	2.42 ± 1.18	nd	1.07 ± 0.88
10	Q-3-glc	18.82	463;301	nd	0.82 ± 0.50	3.62 ± 1.75	2.06 ± 0.76	2.60 ± 0.36	nd	0.42 ± 0.22
11	L-3-glc	19.38	493;331	1.43 ± 0.36	1.38 ± 0.88	1.81 ± 0.03	5.52 ± 0.89	0.87 ± 0.39	nd	nd
12	Q-3-rha	20.14	447;301	nd	nd	nd	nd	0.37 ± 0.17	nd	nd
13	Q-3-xyl	20.85	433;301	1.38 ± 0.81	1.24 ± 0.57	0.57 ± 0.46	1.84 ± 1.28	0.79 ± 0.44	nd	0.52 ± 0.43
14 *	L-3-acglc	21.01	535;331	nd	nd	nd	nd	1.35 ± 0.79	nd	nd
15	Ir-3-glc	21.31	477;315	nd	0.63 ± 0.15	1.56 ± 0.89	2.83 ± 0.52	0.53 ± 0.07	nd	nd
16	K-3-rut	22.16	593;285	nd	nd	nd	1.49 ± 0.73	nd	nd	nd
17	S-3-glc	23.58	507;345	8.09 ± 0.03	1.70 ± 0.03	5.69 ± 1.17	1.59 ± 0.45	1.80 ± 0.48	0.20 ± 0.02	3.10 ± 0.81
	Total			20.42 ± 1.10 ^c^	15.12 ± 1.85 ^bc^	31.12 ± 1.64 ^d^	34.51 ± 1.57 ^d^	20.55 ± 2.77 ^c^	3.51 ± 2.40 ^a^	8.29 ± 1.02 ^ab^
**Flavan-3-ols (mg CE /L)**										
18	epigallocatechin	2.86	305;179,141	nd	0.52 ± 0.16	3.28 ± 0.89	nd	nd	nd	nd
19	catechin	10.88	289	17.25 ± 1.84	7.07 ± 1.14	1.23 ± 0.38	0.63 ± 0.04	2.23 ± 0.37	nd	nd
20	epicatechin	14.66	289	3.94 ± 0.78	1.47 ± 1.02	1.23 ± 0.85	nd	3.21 ± 0.48	nd	nd
21	P2 a	8.92	577;425,289	nd	2.62 ± 1.27	tr	nd	nd	nd	nd
22	P2 b	11.18	577;425,289	4.93 ± 1.68	nd	nd	nd	nd	nd	nd
23	P2 c	13.52	577;425,289	1.21 ± 0.82	0.91 ± 0.71	nd	nd	0.53 ± 0.43	nd	nd
24	P2 d	14.54	577;425,289	3.28 ± 0.94	nd	nd	nd	0.65 ± 0.16	nd	nd
25	P2 e	16.36	577;425,289	8.41 ± 1.79	nd	nd	nd	nd	nd	nd
26	P3 a	12.76	865;577,289	nd	0.63 ± 0.39	nd	nd	nd	nd	nd
27	P3 b	15.78	865;577,289	nd	7.47 ± 1.47	nd	nd	nd	nd	nd
28	P2-glc	16.02	729;577,289	nd	nd	nd	nd	1.96 ± 0.02	nd	nd
	total			39.02 ± 2.71 ^d^	20.69 ± 1.67 ^c^	5.74 ± 0.41 ^b^	0.63 ± 0.04 ^a^	7.41 ± 0.84 ^b^	nd	nd
**Hydroxybenzoic acids (mg GAE /L)**										
29	Gallic acid	0.99	169	8.33 ± 0.12	7.69 ± 0.57	7.79 ± 0.60	8.35 ± 1.62	3.07 ± 0.82	nd	nd
30	Protocatechuic acid	1.87	153	0.97 ± 0.10	0.52 ± 0.23	1.63 ± 0.37	1.15 ± 0.35	2.51 ± 0.83	1.05 ± 0.30	nd
31	Protocatechuic-taric acid	2.18	305;153	nd	nd	nd	1.67 ± 0.66	nd	nd	nd
32	Hexose ester of vanillic acid	4.13	329;167	nd	0.45 ± 0.34	nd	nd	nd	nd	nd
33	p-Hydroxybenzoic acid	5.93	137	nd	1.85 ± 0.42	nd	1.09 ± 0.49	0.92 ± 0.47	2.83 ± 0.56	nd
34	Ethyl gallate	12.99	197;169	1.76 ± 0.27	1.24 ± 0.32	4.81 ± 0.73	2.34 ± 0.49	nd	0.06 ± 0.05	0.62 ± 0.37
	total			11.07 ± 0.29 ^c^	11.74 ± 0.74 ^cd^	14.23 ± 0.24^d^	14.59 ± 1.59 ^d^	6.50 ± 0.46 ^b^	3.94 ± 0.80 ^b^	0.62 ± 0.37 ^a^
**Hydroxycinnamic acids (mg CAE /L)**										
35	cis-Caffeic acid	1.21	179	nd	0.93 ± 0.42	0.68 ± 0.25	0.76 ± 0.27	0.36 ± 0.10	0.82 ± 0.25	1.88 ± 0.30
36	Cinnamic acid	1.28	147	0.28 ± 0.11	nd	nd	nd	0.62 ± 0.13	1.09 ± 0.39	0.58 ± 0.27
37	cis-p-Coumaric acid	2.05	163	nd	nd	nd	1.00 ± 0.43	nd	nd	nd
38	Caftaric acid	3.38	311;179	0.47 ± 0.13	nd	nd	30.62 ± 2.79	29.09 ± 1.36	0.60 ± 0.29	12.09 ± 0.53
39	Cutaric acid	5.99	295;163	nd	nd	2.27 ± 0.72	12.21 ± 1.41	18.16 ± 0.38	nd	13.61 ± 1.37
40	cis-Fertaric acid	10.09	325;193	nd	nd	nd	2.66 ± 0.77	nd	nd	nd
41	trans-Caffeic acid	10.33	179	0.88 ± 0.16	3.17 ± 0.41	1.48 ± 0.44	nd	2.12 ± 0.39	0.49 ± 0.26	1.48 ± 0.25
42	Hexose ester of cis-p-coumaric acid	11.19	325;163	nd	nd	nd	0.24 ± 0.13	nd	nd	nd
43	Hexose ester of trans-p-coumaric acid	13.09	325;163	nd	4.60 ± 0.60	3.49 ± 1.43	3.81 ± 0.58	0.43 ± 0.12	1.12 ± 0.98	0.26 ± 0.12
44	Hexose ester of ferulic acid	13.24	355;193	nd	nd	nd	4.76 ± 0.58	1.05 ± 0.30	nd	0.70 ± 0.26
45	trans-p-Coumaric acid	13.85	163	nd	nd	nd	nd	1.15 ± 0.25	0.43 ± 0.29	1.20 ± 0.31
46	trans-Fertaric acid	14.03	325;193	5.19 ± 0.48	nd	1.09 ± 0.13	nd	1.91 ± 0.57	0.91 ± 0.39	3.66 ± 0.82
	total			6.81 ± 0.34 ^a^	8.70 ± 0.61 ^a^	9.01 ± 1.84 ^a^	56.06 ± 1.31 ^c^	54.88 ± 0.66 ^c^	5.46 ± 0.66 ^a^	35.47 ± 1.52 ^b^
**Stilbenes (mg RE /L)**										
47	cis-Piceid	17.51	389;227	0.42 ± 0.10	0.09 ± 0.02	nd	nd	nd	nd	nd
48	trans-Piceid	21.23	389;227	0.96 ± 0.21	0.13 ± 0.11	tr	0.47 ± 0.08	0.59 ± 0.12	0.50 ± 0.44	0.16 ± 0.05
	total			1.38 ± 0.10 ^b^	0.21 ± 0.09 ^a^	tr	0.47 ± 0.08 ^a^	0.59 ± 0.12 ^a^	0.50 ± 0.44 ^a^	0.16 ± 0.05 ^a^

The abbreviations of the cultivars are the same as Table 1. Values are means of duplicate determination±S.D. Dq, dihydroquercetin; Q, quercetin; K, kaempferol; Ir, isorhamnetin; L, laricitrin; S, syringetin; gal, galactoside; gcn, glucuronide; rha, rhamnoside; caglc, (6-caffeoyl)-glucoside; acglc, (6-acetyl)-glucoside; hex, hexoside; xyl, xyloside; rut, rutinoside; P2, Proanthocyanidin dimer; P3, Proanthocyanidin trimer. * represents tentative assignation. Diffrent letters in each row of total concetrations are significantly different at the 0.05 level according to ANOVA.

#### 2.3.1. Anthocyanin Profiles

The concentrations and compositions of anthocyanins varied considerably between different cultivars and wine types. For red wines, the total concentration of anthocyanins (TAC) was highest in the wine from the American grape ‘Cynthiana’ (186.75 mg ME /L), followed by the French hybrid ‘Chambourcin’ wine (84.59 mg ME /L), while the two European grape wines had TAC of 22.38 mg ME/L for ‘Merlot’ and 24.70 mg ME /L for ‘Cabernet Sauvignon’, the lowest among all the red samples investigated. The rose wine of *V. labrusca* cultivar ‘Catawba’ had 6.81 mg ME /L of TAC, significantly lower than that of the red wines. 

A total of 33 anthocyanins were identified from the coloured (red and rose) wines, including 10 monoglucoside anthocyanins, 10 diglucoside anthocyanins, seven pyranoanthocyanins, three hydroxyphenylpyranoanthocyanins and three anthocyanin-flavanol dimers. It is known that the European grape (*V. vinifera*) wines contain only monoglucoside anthocyanins, while non-*V. vinifera* species have both mono- and di-glucoside anthocyanins. The same results were discovered in our study. In this study, diglucoside anthocyanins were dominant in the American grape derived wines, accounting for 82.91%, 75.62% and 57.42% of TAC in ‘Cynthiana’, ‘Chambourcin’ and ‘Catawba’ wines, respectively. Higher percentages of TAC-derived pigments were seen in ‘Merlot’ and ‘Cabernet Sauvignon’ wines. On average, the pyranoanthocyanin, hydroxyphenylpyranoanthocyanins and anthocyanin-flavanol dimers accounted for 39.46%, 2.31% and 16.35% of TAC in the ‘Merlot’ and ‘Cabernet Sauvignon’ wines, while those accounted for 5.71%, 0.99% and 0 in the other two red wines, ‘Cynthiana’ and ‘Chambourcin’. No derived pigments were detected in the ‘Catawba’ rose wine.

Malvidin derivatives were the dominant anthocyanins in the red wines. Malvidin-3-glucoside was the most abundant anthocyanin in ‘Merlot’ and ‘Cabernet Sauvignon’ wines, followed by malvidin-3-(6-acetyl)-glucoside, malvidin-3-glucoside-pyruvic acid (vitisin A), and malvidin-3-(6-acetyl)-glucoside-pyruvic acid (acetylvitisin A). Malvidin-3,5-diglucoside was the most abundant anthocyanin in the two American red grape wines, at levels seven times higher than the second most important anthocyanin, malvidin-3-glucoside. Among the acylated pigments, acetyl- and coumaryl- anthocyanins were found in red wines. The former was the main type in the European grape wines while the latter were dominant in the American grapes or French hybrid wines. In the rose wine, anthocyanins were mainly comprised of mono- and di-glucosid of cyanidin and delphinidin.

#### 2.3.2. Dihydroflavonols and Flavonol Profiles

The dihydroflavonols convert to the flavonols through the formation of a double bond between the positions of C-2 and C-3 in the molecules by the flavonol synthase [28], so the two kinds of phenolic compounds can be bracketed together [29,30]. Total concentrations of dihydroflavonols and flavonols (TFO) varied widely among the wines analyzed, ranging from 4 mg QE /L to 35 mg QE /L. American grape ‘Cynthiana’ (34.51 mg QE /L) and hybrid ‘Chambourcin’ (31.12 mg QE /L) had significantly higher flavonol concentrations than the two European grape red wines (20.42 and 15.12 mg QE /L) and the ‘Catawba’ rose wine (20.55 mg QE /L). As expected, the two white wines (‘Villard blanc’ and ‘Noah’) had the lowest TFO (8.29 and 3.51 QE mg/L).

There were four dihydroflavonols identified, all of which were dihydroquercetin derivatives. Thirteen flavonols were observed in all the wine samples. In general, dihydroquercetin and quercetin derivatives were the most important types, with varying percentages among different wines. The former was dominant in the European grape wines (46.60% for ‘Merlot’ and 56.26% for ‘Carbernet Sauvignon’) and *V. labrusca* ‘Catawba’ (42.80%), while the latter was the main type found in the American grape ‘Cynthiana’ (42.12%) and ‘Noah’ (52.94%), and the French hybrid ‘Chambourcin’ (50.37%) and ‘Villard Blanc’ (94.25%). In addition, the only syringetin derivative, syringetin-3-*O*-glucoside was also a common flavonol found in all the wines analyzed.

Among all the dihydroflavonol and flavonal compounds, peaks 5 and 14 were tentatively assigned as quercetin-3-(6-caffeoyl)-glucoside and laricitrin-3-(6-acetyl)-glucoside, respectively, on the basis of their MS data. A signal at *m/z* 301 was found in the mass spectrum of peak 5, which indicated a quercetin-type flavonol, together with a signal at 625 m/z units that can be associated with the molecular ion ([M–H]^–^). In the same way, the mass spectrum of peak 14 showed the signals at 331 *m/z* units (laricitrin-type flavonol) and 535 *m/z* units ([M–H]^–^). However, there is a dispute about the existence of acylated flavonols in grapes and wines [31,32], and the assignation proposed must be further confirmed by NMR spectroscopy.

#### 2.3.3. Flavan-3-ol Profiles

There were significant differences in total flavon-3-ol concentrations (TFA). *V. vinifera* ‘Merlot’ wine possessed the highest level of TFA (39.02 mg CE /L), followed by the other *V. vinifera* cultivar ‘Carbernet Sauvignon’ wine (20.69 mg CE /L). Low concentrations of TFA were found in *V. labrusca* ‘Catawba’ wine, the French hybrid ‘Chambourcin’, and the American grape ‘Cynthiana’ (7.41, 5.74 and 0.63 mg CE /L, respectively). No flavon-3-ols were detected in the two white wines made from the French hybrid “Villard Blanc” and *V. labrusca* “Noah”.

Among the 11 detected flavan-3-ol compounds, three were monomers (epigallocatechin, catechin and epicatechin), five were dimers, two were trimers, and one was a dimer-glucoside. The monomers and oligomers were relatively rich in the two *V. vinifera* red wines, accounting for half of TFA, respectively. On the contrary, the flavan-3-ols obtained in the other two red wines from American grapes or hybrids were mostly composed of monomers, and only a trace of procyanidin dimer was found in the French hybrid ‘Chambourcin’ wine. In the rose wine, the monomers and oligomers accounted for 73.48% and 26.50% of TFA, and the oligomers were comprised of two procyanidin dimers and one dimer-glucoside. Catechin was common among the individual flavanols in the coloured wines.

#### 2.3.4. Phenolic Acid Profiles

There are two kinds of phenolic acids, hydroxybenzoic acids and hydroxycinnamic acids, in wines on the basis of their molecular structures. Interestingly, the total concentrations of hydroxybenzoic acids (TBA) were varied according to different wine types. The highest levels were found in the two red wines made from American grapes or hybrids (14.23 and 14.59 mg GAE /L), followed by the two red *V. vinifera* grape wines (11.07 and 11.74 mg GAE /L), while the lowest were found in the two white wines (0.62 and 3.94 mg GAE /L), TBA of the rose wine lay between (6.50 mg GAE /L) the red and white wines. For the individuals, six compounds were detected, and gallic acid took up the highest percentage of TBA in all the wines except for two white wines, ranging from 47% to 75%.

The distribution and concentrations of total hydroxycinnamic acid (TCA) in all the wines was very different from the hydroxybenzoic acids, which varied greatly among different cultivars. The wines made from American grapes ‘Cynthiana’ and ‘Catawba’ had the highest TCA (56.06 and 54.88 mg CAE /L), followed by the white wine made from *V. labrusca* ‘Noah’ (35.47 mg CAE /L). Less than 10 mg CAE /L of TCA were found in other wine samples in this study. A total of 12 hydroxycinnamic acids were identified, and mainly contained *p*-coumaric, caffeic and ferulic acids, as well as their tartaric and hexose esters.

#### 2.3.5. Stilbene Profiles

The total stilbene concentrations (TS) in all these wines were relatively low, ranging from 1.38 RE mg/L for ‘Merlot’ wine to a trace for ‘Chambourcin’ wine. Two stilbenes (*cis*- and *trans*-piceid) were detected. *trans*-Piceid was more common in all the samples.

### 2.4. Antioxidant Capacity

Ferric reducing antioxidant power (FRAP), which has been proven to correlate well with other measurement methods [11,12], was used to estimate the antioxidant capability of the wine samples. FRAP varied with different wine types and cultivars. The FRAP values ranged from 2.91 mmol TE/L to 8.47 mmol TE/L for red wines, 1.35 mmol TE/L and 2.17 mmol TE/L for white wines, and 2.72 mmol TE/L for the rose wine (Table 2). The highest FRAP value was found in the red wine of the American grape ‘Cynthiana’, while the lowest was in the white wine from the French hybrid ‘Villard Blanc’.

Correlations of individual and total phenols to the wine FRAP were also estimated. TPC (r^2^ = 0.996), TAC (r^2^ = 0.984), TFO (r^2^ = 0.850) and TBA (r^2^ = 0.797) exhibited significant correlations to FRAP. A strong correlation between most anthocyanin types and FRAP was found, including monoglucoside anthocyanins (r^2^ = 0.949), diglucside anthocyanins (r^2^ = 0.965), pyranoanthocyanins (r^2^ = 0.943), hydroxyphenyl-pyranoanthocyanins (r^2^ = 0.852) and all the five aglycone-based anthocyanins (malvidin, r^2^ = 0.972; delphinidin, r^2^ = 0.865; petunidin, r^2^ = 0.919; cyanidin, r^2^ = 0.812; peonidin, r^2^ = 0.878). Among the different flavonols, FRAP was remarkably well correlated to quercetin, kaempferol, laricitrin and isorhamnetin derivates (r^2^ = 0.795, 0.871, 0.925 and 0.970, respectively). Only the gallic acid out of the phenolic acids had an obvious correlation to FRAP (r^2^ = 0.823).

## 3. Discussion

The phenolic assays varied among different wine types. More phenolic compounds were extracted from the pomace during the maceration and fermentation for red wines, while fewer phenols were extracted from the white wines due to the rapid pressing to separate the must from the solid matter [33,34]. For most rose wines, maceration times are shortened to give the wines a rose or light pink colour [35,36]. We made the rose wine from the American grape ‘Catawba’ using the same process as for the red wines. The rose colour was obtained by the low concentration and different composition of anthocyanins. Our results indicated that the TPC and FRAP of the red wines were significantly higher than those observed in the white wines, and that the rose wine was in between the red and white wines, which is in agreement with previously published reports [12,37]. In addition, low flavonols, low gallic acid and non flavon-3-ols in the white wines were also due to lack of phenolic extraction from the must.

The phenolic compounds contained within the grape berries are extracted into wines, mostly from the berry skins. The biosynthesis of phenolics is strictly controlled by the corresponding enzyme activities in the respective biosynthetic pathways. At the same time, the wine phenolic profile from a given cultivar can reflect its genetic potential [38]. Among the four red wines, the wine from the American grape ‘Cynthiana’ contained the most abundant anthocynins and flavonols, followed by the ‘Chambourcin’ wine. The ‘Merlot’ and ‘Cabernet Sauvignon’ wines had the lowest anthocynins and flavonol concentrations. The flavan-3-ol concentrations in these red wines were in reverse order. That may be due to the reason that anthocynins, flavonols and flavan-3-ols share a series of steps of the upstream pathways [28,30] and genotypes of different species/cultivars have inclinations towards different downstream biosynthetic pathways.

For anthocyanins, the concentrations in red wines from the American grapes and hybrids ‘Cynthiana’ and ‘Chambourcin’ were significantly higher than those in the two European ‘Merlot’ and ‘Cabernet Sauvignon’ red wines. At least two major differences were observed in the anthocyanin compositions of red wines made from the European and the North American grapes. First, diglucoside anthocyanins were the main anthocyanin type in the wines from American grapes, which were more stable than the monoglucoside anthocyanins. The monoglucoside anthocyanins were liable to react with other molecules, such as pyruvic acid and acetaldehyde, during the processes of fermentation and aging. Second, low flavanol concentrations in non-*V. vinifera* wines reduced the chance of the formation of anthocyanin-flavanol adducts. The derived pigments (pyranoanthocyanins, hydroxyphenylpyranoanthocyanins and anthocyanin-flavanol dimers), the key pigments for the colour stability of the aged *V. vinifera* wines, less effect by external storage environment, and diglucoside anthocyanins were easier to brown than monoglucoside anthocyanins, which is the reason why the colour of aged *V. vinifera* wines is more stable than that of aged wines from other species grapes. Therefore, it is necessary to further investigate how to improve colour stability and aging potential of the American grape wines that show a high concentration of diglucoside anthocyanins, a low concentration of derived pigments and the main copigment, flavanols.

There are some disputes about the pedigree of ‘Catawba’ and ‘Cynthiana’. Although ‘Catawba’ are usually categorized as a *V. labrusca* cultivar [39], most experts suspect that it is a hybrid of the native American *V. labrusca* and another *Vitis* species, maybe even *V. vinifera*. In our results, the flavonol concentrations and compositions in this wine were similar to the *V. vinifera* wines, indicating the possibility of hybridization with *V. vinifera.* ‘Cynthiana’ is believed to be largely derived from *V. aestivalis* [40], but is also likely to be a cross of one or more native American varieties and one *V. vinifera* grape. Our results support the former view because the phenolic characteristics of the ‘Cynthiana’ wine were very different from those found in the *V. vinifera* wines. 

The phenolic concentration of wines can be attributed to their antioxidant capacities. We found a significant correlation between antioxidant activity and spectrophotometrically measured total phenolic contents, in line with previous reports [41,42]. However, various conclusions were found about the correlation between specific phenolic compounds and their antioxidant activity. Alén-Ruiz *et al.* obtained high correlations of total flavonols and acylated anthocyanins with anti-radical activity in the wines stored for 3 months [39]. Vrček *et al.* showed that gallic acid, caffeic acid, *p*-coumaric acid and resveratrol significantly correlated to the antioxidant activity of wine *in vitro* [11]. De Quirós *et al.* found that the antioxidant activities of white wines had a good correlation with quercetin contents and a reasonable correlation with rutin and procyanidin B1 [43]. Makris *et al.* concluded that the antioxidant potency of wines was correlated with the concentrations of hydroxycinnamates and flavanols [42]. In our study, antioxidant activity was strongly correlated to the two major classes of wine phenols, anthocyanins and flavonols, but poorly correlated to the flavanols, stilbenes and most of the phenolic acids.

We also realized that the total concentrations of phenolics and anthocyanins for the red and rose wines were remarkably lower than those found in the previous reports [44,45]. That was because winemaking technique by hand for a small amount in the laboratory scale was easier than that in mechanized production, for operations such as stirring and mixing of must with pomace in the processes of alcoholic fermentation, but this couldn’t influence the comparison of the phenolic characteristics of the seven wine samples, since the cultivation climate, viticulture practice and winemaking method in this study were the same. Due to the samples from only one vintage and one viticultural region, the results in this study were preliminary and we would continue to further research on the quality characteristics of these or more North American wine grapes in the following vintages. Then some wine grape cultivars with good adaptability and high quality would be picked out to increase the cultivation and improve their winemaking techniques for a large amount.

## 4. Experimental

### 4.1. Materials

Five American wine grape cultivars (‘Cynthiana’, ‘Chambourcin’, ‘Catawba’, ‘Villard Blanc’, ‘Noah’) and two traditional European grapes (‘Merlot’ and ‘Cabernet Sauvignon’) were harvested at their stages of optimum maturity in the summer of 2009. American grapes were grown in the experimental vineyards of China Agricultural University in Beijing, while European grapes were grown in nearby Ji County, Tianjing. This viticulture area belongs to the continental monsoon climate zone with sub-humid warm temperature during the fruit development and ripening season.

In order to minimize the effects of vinification technology, all the wine samples examined were produced and stored under lab conditions. The winemaking method for a small amount [46] was used with some modifications (Wang, personal communication) in this study. Briefly, the grape berries were destemmed, crushed, and poured into the glass containers (10 L). In order to prevent oxidation and inhibit bacteria growth, 0.2‰ HSO_3_ was added into the containers. For red and rose wines, the action of HSO_3_ was maintained for 30 minutes, and then the must was inoculated with live yeast (BM45, Lallemand Co.) and fermented for 7 days at 20–28 °C. The pomace was separated from the must, and the fermented juices were racked into the glass containers with a venting device, which allowed only the gas to vent from the containers and kept the outside air off the containers. In the containers, the yeasts would finished the alcoholic fermentation for reducing the residual sugar and further stabilised in a period of 35 days at 18 °C. For white wines, the must was added with HSO_3_ for 12 hours before pressing. Juice was fermented and further stabilised at 18 °C for 40 days. Then the young wines were aged for 6 months at 18 °C and racked for two times. Each wine sample from one fermented containers was considered as one replicate resulting in two replications for each grape cultivars.

### 4.2. Chemicals and Standards

Folin and Ciocalteu’s phenol reagent (2 N) and 2,4,6-tripyridyl-*s*-triazine (TPTZ, ≥99%) were obtained from Sigma–Aldrich (St. Louis, MO, USA). 6-Hydroxy-2,5,7,8-tetramethylchroman-2-carboxylic acid (Trolox, ≥99%) was obtained from Alexis (Axxora, Switzerland). HPLC grade reagents, methanol, formic acid, acetic acid and acetonitrile were obtained from Fisher Co. (Fairlawn, NJ, USA). All other chemicals and reagents were analytical grade and purchased from Lanyi Co. (Beijing, China). The standards, caffeic acid, gallic aid, resveratrol, malvidin-3-*O*-glucoside, quercetin and catechin were all purchased from Sigma–Aldrich.

### 4.3. Determination of Oenological Parameters

Two 100-berry batches were sampled from at least 50 cluster selections at similar positions of at least six whole vine selections. The two parallel grape samples were weighed, and their juices were subjected to the determinations of total soluble solids (TSS) and titratable acidity (TA) according to the International Organisation of Vine and Wine (OIV) methods [47]. The alcoholic strength, titrable acidity (TA), volatile acidities (VA) and residual sugar (RS) of wine samples were assayed using OIV methods [47].

### 4.4. Determination of Total Phenolic Concentration

The total phenolic concentrations (TPC) among these wines were determined by the Folin-Ciocalteu colourimetric method with slight modification [48,49]. Briefly, all samples were diluted 20 times in order to make readings in the standard curve ranges of 50–1,000 µg gallic acid/mL, then Folin-Ciocalteu reagent and sodium carbonate were successively added. The solution was reacted in a water base (40 °C) for 30 min. The standard curve was constructed with gallic acid. Absorbance was assayed using a UV-2800 spectrometer (UNICO, New York, NY, USA) and converted into TPC expressed as milligrams of gallic acid equivalence (GAE)/g of dry matter.

### 4.5. Preparation of Samples for HPLC-MS/MS

The wines were filtered through a 0.45 µm inorganic membrane and used directly for anthocyanin analysis. For non-anthocyanin assays, samples were extracted with 100 mL ethyl acetate thrice [30] and then filtered through 0.22 µm organic membranes.

### 4.6. Qualitative Analysis of Phenolic Compounds by HPLC-MS/MS

Analyses of anthocyanins were performed on an Agilent 1100 series LC-MSD trap VL, equipped with a G1379A Degasser, a G1311A QuatPump, a G1313A ALS, a G1316A Column, a G1315B DAD and a Kromasil-C18 column (250 × 4 mm, 6.5 µm). The mobile phase was: 6% (v/v) acetonitrile containing 2% (v/v) formic acid as solvent A, and 54% (v/v) acetonitrile containing 2% (v/v) formic acid as solvent B. The gradient was as follows: 0–1 min, 10% B; 2–17 min, 10%–25% B; 18–20 min, 25% B; 21–30 min, 25%–40% B; 31–35, 40%–70% B; 36–40 min, 70%–100% B. The flow rate was 1.0 mL min^−1^ and the column temperature was set at 50 °C. The injection volume was 30 µL and the detection wavelength was 525 nm. Electrospray ionization (ESI) with a positive ion model, 35 psi nebulizer pressure, 10 mL min^−1^ dry gas flow rate, 325 °C dry gas temperature, and 100–1,000 *m/z* scan range were used for MS analysis [30].

Non-anthocyanins analyses were performed with an Agilent 1200 series instrument equipped with a G1322A Degasser, a G1312B Binary pump, a G1367C HiP-ALS, a G1316B TCC, a G1314C VWD, and a ZORBAX SB-C18 column (3 × 50 mm, 1.8 µm). The mobile phase A was a water solution with 1% acetic acid and the mobile phase B was an acetonitrile solution with 1% acetic acid. The gradient was as follows: 0–10 min, 5%–8% B; 11–18 min, 8%–10% B; 19–40 min, 10%–15% B; 41–50 min, 15%–20% B; 51–53 min, 20%–30% B; 54–58 min, 30%–50% B; 59–62 min, 50%–100% B; 63–66 min, 100% B, with a flow rate at 1.0 mL/min, Injection volume was 2 µL, the detection wavelength was 280 nm, and the column temperature was 25 °C. MS analysis was used ESI, negative ion model, 35 psi nebulizer pressure, 10 mL min^−1^ dry gas flow rate, 325 °C dry gas temperature, and 100–1,000 *m/z* scan range [30].

### 4.7. Quantification of Phenolic Compounds

Anthocyanins, flavonols, flavan-3-ols, hydroxybenzoic acids, hydroxycinnamic acids and stilbenes were respectively expressed as micrograms of malvidin-3-*O*-glucoside (ME), quercetin equivalence (QE), catechin equivalence (CE), gallic acid equivalence (GAE), caffeic acid equivalence (CAE), and resveratrol equivalence (RE) /L of wine.

### 4.8. Determination of Antioxidant Capacity by Reducing Power

The antioxidant capability of wines was determined through the method of ferric reducing antioxidant power (FRAP) [50]. Different concentrations (100–1000 µM) of Trolox were used to measure the standard curve. The values of FRAP were expressed as Trolox equivalent antioxidant activity (µM TE/g DM).

### 4.9. Statistical Analysis

All experimental data were expressed as means ± standard deviations (S.D.) of duplicates and subjected to one-way analysis of variance (ANOVA) with SPSS 16.0 (SPSS Inc.) at 95% confidence level. The correlations between major phenolic compounds and FRAP were analyzed using SPSS 16.0 (SPSS Inc.).

## 5. Conclusions

The phenolic characteristics of wines varied largely depending on different wine types and cultivars/species. The red wines had significantly higher total phenolic concentrations and antioxidant capacities than the white wines, and those in the rose wine lay between the red and white wines. In the red wines, the two wines from American *Vitis* or hybrids grapes ‘Cynthiana’ and ‘Chambourcin’ exhibited more abundant total phenols, anthocyanins, flavonols, phenolic acids and antioxidant capacity than the two *V. vinifera* wines from ‘Merlot’ and ‘Cabernet Sauvignon’, while the case of flavon-3-ols was just the reverse. The *V. vinifera* ‘Merlot’ wine had the most stilbenes among all the wines investigated. For the phenolic compositions, diglucoside derivatives were the dominant anthocyanins in the coloured wines from American *Vitis* or hybrids, while only monoglucoside derivatives existed in the *V. vinifera* wines; the main flavonol type was dihydroquercetin derivatives in most American *Vitis* or hybrids wines, while quercetin derivatives were main in the the two *V. vinifera* wines and the rose wine from American grape ‘Catawba’; flavan-3-ols were mainly comprised of monomers in the wines from coloured American grapes, while both monomers and polymers were important in the *V. vinifera* wines. The antioxidant activity with ferric reducing antioxidant power was found a significant correlation with total phenols (r^2^ = 0.996), total anthocyanins (r^2^ = 0.984), total flavonols (r^2^ = 0.850) and gallic acid (r^2^ = 0.797).
